# P001. Trigeminal neuralgia-like symptoms: an unusual case

**DOI:** 10.1186/1129-2377-16-S1-A102

**Published:** 2015-09-28

**Authors:** Andrea Giorgetti, Patrizia Perrone, Maria V Calloni, Serena Leva, Francesco Muscia, Lucia Politini, Emilio Vecchio

**Affiliations:** UO Neurologia-Stroke Unit, AO Ospedale Civile di Legnano, Legnano, Italy

## Introduction

Lymphoma of the central nervous system[1] accounts for 2% of cerebral tumors; it is typically located in the supratentorial and periventricular white matter; extra-axial localization is rare. When found in the cerebellopontine angle or the Meckel cave[2], the differential diagnosis includes meningioma, trigeminal neurinoma or epidermoid carcinoma. Correlated painful symptoms are often atypical, mimicking trigeminal neuralgia, cluster headache or trigeminal autonomic cephalgias (TACs). Recommended therapy involves: chemotherapy with cyclophosphamide, high dose cytarabine, steroid (dexamethasone), etoposide, and rituximab (CHASER) followed by whole-brain irradiation.

We describe the case of a young man who came to our observation for excruciating headache related to mediastinal lymphoma with bilateral infiltration of the the ganglion of Gasser.

## Case report

A 30-year-old man came to our attention suffering for the past 3 months of left fronto-orbital-zygomatic headache with intense pain, mainly nocturnal, subcontinuous, initially with nasal congestion and conjunctival injection, unresponsive to FANS, triptans and oxygen therapy. Brain MRI showed a lesion in the left Meckel cave suggestive of trigeminal neuroma. The patient was treated with carbamazepine and steroids. Radiosurgical treatment was advised. Waiting for surgery, the patient was admitted to our department because of inadequate pain control. The neurological examination and serological tests were normal. Follow-up brain MRI (two months later) revealed the presence of pathologic tissue with homogeneous enhancement in both ganglia of Gasser cisterna along the course of the trigeminal nerve, associated with thickening of the dural surface and adjacent the temporal pole, suggesting granulomatous or lymphoproliferative disease. Cerebral spinal fluid (CSF) examination showed hyperproteinorrachia and non-neoplastic cells. The chest and abdominal CT showed a mediastinal and pancreatic mass, confirmed by PET total body. Histological examination of mediastinal lesion confirmed diffuse large B cell lymphoma. The patient is now in treatment with high-dose MTX infusion.

## Discussion

Our case emphasises the need for proper and timely diagnosis of the “trigeminal neuralgia-like” symptoms. With a lesion in the Meckel cave, biopsy is mandatory for a more precise diagnosis and targeted therapy[3].

Written informed consent to publication was obtained from the patient(s).
